# A qualitative study to explore views of patients’, carers’ and mental health professionals’ to inform cultural adaptation of CBT for psychosis (CBTp) in China

**DOI:** 10.1186/s12888-017-1290-6

**Published:** 2017-04-08

**Authors:** Weihui Li, Li Zhang, Xuerong Luo, Bangshan Liu, Zhipeng Liu, Fang Lin, Zhiling Liu, Yuhuan Xie, Melissa Hudson, Shanaya Rathod, David Kingdon, Nusrat Husain, Xudong Liu, Muhammad Ayub, Farooq Naeem

**Affiliations:** 1 Mental Health Institute of the Second Xiangya Hospital, Central South University, Changsha, Hunan 410011 China; 2 National Clinical Research Center for Mental Disorders, Changsha, China; 3 National Technology Institute on Mental Disorders, Changsha, China; 4 Hunan Key Laboratory of Psychiatry and Mental Health, Changsha, China; 5 Jiangxi mental health center, Changsha, China; 6 Xiangyang Anding Hospital, Changsha, China; 7 Chuxiong Prefecture’s Mental Hospital, Changsha, China; 8 Department of Psychiatry, Queens University, 191 Portsmouth Avenue, Kingston, ON K7M 8A6 Canada; 9 Southern Health NHS Foundation Trust, Clinical Trials Facility, Tom Rudd Unit, Moorgreen Hospital, Southampton, SO30 3JB UK; 10 The University of Southampton, Southampton, UK; 11 The University of Manchester, Manchester, UK

**Keywords:** Culture, Adaptation, Cognitive therapy, Psychosis, Schizophrenia, China

## Abstract

**Background:**

The evidence for effectiveness of Cognitive Behaviour Therapy (CBT) is robust and the national organizations in the United Kingdom and the United States recommend its use. It is not utilized to its full potential in low and middle-income countries. Adaptation of CBT treatment to the target culture may facilitate its uptake.

This study explored views of patients with schizophrenia, their caregivers, and mental health professionals for the purpose of cultural adaptation of CBT.

**Method:**

The project was conducted in a teaching hospital in China. Systematic content and question analysis were the techniques we used to analyse the data generated in a series of qualitative interviews (N 45) in China. After identification of emerging themes and categories we compared and contrasted the themes across different interviews recursively. Triangulation of themes and concepts was undertaken to compare further and contrast the data from the different participating groups.

**Results:**

This work highlighted the barriers in therapy as well as opportunities for use of CBT in that environment. Patients and their carers in China use a bio-psycho-spiritual-social model of illness. CBT is not commonly used to help those with schizophrenia in China.

**Conclusions:**

This study will facilitate the therapists using CBT for people with psychosis in China. These results require to be tested in clinical trials.

**Electronic supplementary material:**

The online version of this article (doi:10.1186/s12888-017-1290-6) contains supplementary material, which is available to authorized users.

## Background

The evidence to support the effectiveness of CBT for psychosis (CBTp) in Low & Middle-Income Countries (LAMIC) is emerging [1–5], but it is rarely available in these countries. One possible barrier to its use might be a lack of adaptation to address local barriers [6]. Cognitive behaviour therapy was developed in the West. Cultural adaptation of CBTp has been recommended for its application in non-western cultures [7–9].

There has been a noticeable improvement of the mental health system in China since the first National Mental Health Plan issued in April 2002. However, like many other LAMICs, the health system harbours a large service gap in the form of a severe shortage of mental health professionals and resources [10]. The most underdeveloped area in this aspect is the integration of psychosocial interventions in the mental health system. There is a recent drive to make evidence-based therapies, particularly CBT available instead of developing indigenous treatments, as CBT might be compatible with Chinese culture if appropriately adapted [11].

Chinese culture is influenced mainly by Confucianism and Taoism, two different philosophies. Confucianism can be considered as a system of social and ethical philosophy and is believed to have had a powerful influence on Chinese behaviour and social structure for the past 2000 years. The central teachings of Confucianism—which include filial piety, respect for familial and social hierarchy, discouragement of self-centeredness, emphasis on academic achievement, and the importance of interpersonal harmony—are considered principles for social interactions and have profound influences on cognitions and behaviors of Chinese individuals [12]. However, the Taoism advocates a life of complete simplicity, being connected with nature, and of non-interference with the course of natural events. Most Chinese seem to be able to combine these two philosophies with great ease to guide or rationalize their thinking and behaviours.

Most of the literature on the use of CBT for Chinese population comes from the United States of America, Australia, and Hong Kong. It has been suggested that the Chinese cultural norms and philosophy of CBT are in perfect harmony with each other [12]. It has also been argued that the Chinese culture places special emphasis on logical thinking. A well-known Chinese proverb says, “Originally there is no disturbance in the world, but people make themselves feel worried” [13] a notion that precisely mirrors the basic concept of CBT. Similarly, Hodges and Oei [11] suggest a substantial degree of compatibility between the Chinese culture and CBT philosophy. They also argue that instead of developing indigenized therapies it would be better to adapt an evidence-based intervention like CBT for the Chinese people. Authors in this area warrant caution and have advised modifications to improve therapeutic outcomes [8].

In our previous work we have used mixed methods approach for cultural adaptation of CBT for both depression and psychosis. Numerous adjustemnts were required to improve accesability, acceptability and efficiency of CBT [1, 4, 14–16]. Based on this work we have developed a methodology to culturally adapt CBTp that has been described in details elsewhere [15, 17, 18]. This paper reports findings from a qualitative study in which we explored the views and opinions of the mental health professionals, patients and their caregivers to inform cultural adaptation of CBT for the Chinese population.

## Aims and objectives

The primary aim of this study was to gather information that can be used to adapt CBTp in China through exploring the views of patients with schizophrenia, their carers, and the mental health professionals. To achieve this broad aim, we conducted interviews with (a) the patients with psychosis and their carers; to explore their views about psychosis, its causes, and the treatment; and (b) mental health professionals helping patients with schizophrenia to examine their experience with this group.

## Method

### Interviewers

Interviews were conducted by one Associate Professor and five residents in their final year training in Psychiatry (average duration of working in psychiatry =8.8 years, range = 4 to 18 years) in Chinese and then transcribed and translated in English as and when they became available. All the interviewers had an MD and had received training in research and psychiatry. Two interviewers were male, with an average age of 31.8 years (range = 26 to 40 years). Interviewers were not directly involved in the care of patients they interviewed, to ensure that the patients are free to express their views. They received training and supervision from the last authors who have vast experience of qualitative research in this area. For example, based on our experience of conducting qualitative research from other countries with a similar background, interviewers tried to assure patients that their disclosure of non-medical treatments will be acceptable, as we noticed medical professionals in these cultures are not accepting of treatments that are not based on a medical model. Regular supervision also addressed other biases, for example, the status gap with patients. Interviewers provided details of the study, gave participants the opportunity to ask any questions they had and sought informed consent before each interview.

### Study design

This qualitative work was underpinned by the principles underlying an ethnographic approach [19, 20]. This approach to data collection allowed us to understand the focus of people participating in the study within their cultural context [21]. The data were analyzed using content analysis.

### Participants and settings

The particpants in the study were recruited by using purposive sampling. Participants were invited by the research team in face to face contacts. They were given information about the study, and those who agreed were invited to the interview. No records were kept of those who refused to participate in the study, as the numbers were too small. All the interviews were conducted in a healthcare facility. The mental health professionals were interviewed at their offices. The interviews with patients and their carers were conducted in the outpatient clinics.

Based on our previous experience we envisaged that we would require between 8 and 12 participants in each group to get a comprehensive picture. We, therefore, decided to recruit 15 participants in each group. We interviewed 15 patients with a diagnosis of schizophrenia/schizoaffective disorder or delusional disorder using ICD-10, RDC (International Classification of Diseases, 10th edition, Diagnostic Criteria for Research) from outpatient clinics of a psychiatry department with illness lasting for a minimum of one year. We excluded patients with severe illness, disruptive behaviour, intellectual disability or serious substance misuse problems from the study. We enlisted 15 caregivers accompanying the patients in the outpatient clinics of participating psychiatry departments, because of the vital role they play in decision making process in this culture. Finally, we interviewed 15 mental health professionals. We wanted to understand their experience of helping patients with schizophrenia and their carers. To help improve access and to adapt CBT, we engaged mental health professionals who practiced CBT or, at least, were aware of the basic concepts of CBT. Table 1 describes characteristics of participants.

**Table 1:** Characteristics of the participants

Patients (15)	
Age	Mean = 22 years (range = 16–34)
Gender	Male = 8 (53.3%), female = 7 (46.7%)
Duration of illness	Mean = 53.6 months (range = 1–180 months)
Education	Mean = 10.5 years (range = 6–19 years)
Marital status	Married = 2 (13.3%), single 13(86.7%)
Diagnoses	Schizophrenia = 15(100%)
Urban/rural	Urban = 11(73.3%), rural =4(26.7%)
Carers (15)	
Age	Mean = 43.3 years (range = 29–53)
Gender	Male = 3(20.0%) female = 12(80.0%)
Marital status	Married = 15(100%)
Relation with patient	Spouse =0(0%), parent = 12 (80%) sibling =2 (13.3%) relatives(aunt) =1 (6.7%)
Urban/rural	Urban = 9(60.0%) rural =6(40.0%)
Psychiatrists (15)	
Age	Mean = 32.1 years (range = 26-36 years)
Gender	Gender male 7 (46.7%), females 8 (53.3%)
Experience in psychiatry	Mean = 7.3 years (range = 3-15 years)

### Collection of data

We had developed and used semi-structured interviews for cultural adaptation of CBT previously [6, 18]. We found them effective [18]. The semi-structured interviews with all the three groups were conducted individually in face to face contact. These semi-structured interviews have open-ended questions with prompts ﻿(Additional files 1, 2 and 3)﻿. These interviews help the researcher explore areas that have already been considered to be vital for cultural adaptation of CBTp. Table 2 summarizes themes explored through interviews. These semi-structured interview guides can be obtained from authors.

**Table 2:** Summary of themes explored through interviews with stakeholders

Patients	
Their knowledge of illness, its causes and its treatment	
Patients perception of effect of the disease on their lives	
Presenting problems and care pathways	
Their experience of modern and traditional methods of help.	
Their understanding of the ideal treatment. Their knowledge of psychotherapy/CBT	
Carers	
Their knowledge of the illness	
Their understanding of cause and treatment of diseases.	
The reasons for bringing patient to the hospital	
Their expectations from treatment and their experience of treatment	
Their knowledge and experience of traditional methods of help	
Their understanding of psychotherapy/CBT	
Psychologists & Psychiatrists	
Their experience of working with schizophrenic patients and their families	
Their perception of pathways of care	
Their experience and expectations from psychotherapy for this patient group	
What are the barriers in providing therapy?	
What is helpful in providing therapy for this group	
Which techniques are useful	
Do they think therapy needs adapting?	
To explore their opinion on effect of culture and other factors on therapy	

The interviews were 60 to 90 min in duration and were audio-recorded. Each interviews was transcribed immediately after completion and ten translated by two authors (BL and LX). These transcripts and translations were randomly assessed by two senior authors (WL&YX) for validity. We acquired contact telephone numbers from the participants and asked their consent to contact them for further clarification of any point raised during the interview. Anonymity and confidentiality were assured. Data was not shared beyond the research team. Any non-verbal communication and behaviours were recorded in the field notes by researchers. We used telephone and video conferencing for communication with and supervision and support to the field team during the study. The interviews were conducted between January and June 2015. We sent the interview scripts to a randomly selected sub-sample of the participants for comment (5 participants from each group), verification and to clarify any issues that arose during the analysis stage. Finally, the results once compiled were shared with all participants.

### Analysis of data

The data were analyzed by systematic content and question analysis [22]. The researcher immersed herself in the data by carefully reading the interview scripts multiple times and identifying emerging themes and categories [20]. Each interviewer started analyzing the interviews as and when they were conducted. We followed the principle of “emergent design” when the issues requiring further exploration were raised by the respondent [23]. These issues were then tested appropriately in subsequent interviews with the participants. We also contacted participants by telephone for clarification of areas of uncertainty when the data were analyzed.

Each interviewee was assigned a numbers for the purpose of the transcription and reporting. The data were primarily descriptive, with most themes emerging in response to the interviews. As predicted saturation point was reached after 10–12 interviews. However, we completed the total number of interviews (*n* = 15) to ensure completeness of data. We adopted an elaborate method of coding. Two teams with three members in each team, one located in China and the second based in Canada coded data separately for the Chinese and the English version of the transcripts. This was to improve the reliability of the analysis and to ensure that the translation is working. The last two authors (MA&FN) supervised the two teams. Finally, the data were reorganized into wider themes (for example, barriers to therapy) and categories (for example, financial burden) and written for this article. Themes were identified in advance based on our previous work which had formed the basis of semi-structured interview.

## Results

Five Chinese interviewers conducted a total of 45 interviews. We organized findings from the interviews under different themes and subthemes. Themes were predetermined from our previous work, and hence the analyses were restricted to those themes. Themes from various groups of participants are presented where possible under headings for the sake of comparison. However, as the themes were predetermined, it was not possible to compare themes across the group of participants. We have also tried to follow loosely the framework we originally developed in analyzing these themes [15].

### Culture and related issues

#### The need for cultural adaptation

Nearly all the health professionals highlighted the importance of cultural and spiritual values and norms in delivering therapy. They emphasized the need for adaptation of therapy according to local needs, taking into account cultural and spiritual factors. In their view cultural beliefs influence patients’ understanding of illness its causes and the treatment.

*Yes, I have to make changes in Western techniques for their use in China (mental health professional 1). Yes, it (CBT) can be employed in China, but it needs to be adapted (mental health professional 3). The therapy I used was adjusted to the characteristics, culture, customs and tradition of people in the East. Therapies, created in West, are a problem to apply for local patients. It should be adapted to our national conditions and (to the) cultural characteristics of Chinese (mental health professional 5). People’s thoughts, emotions, and behaviours are all deeply influenced by religion and culture (mental health professional 11).*

#### Importance of language & communication

Almost all the mental health professionals talked about translation and importance of language that can be understood by the patients. Nearly all the mental health professionals said that they do not use assertiveness techniques. When talking about the language, they all insisted on an adaptation of language, rather than its translation.

*The language used in psychotherapy should be adapted to local culture (mental health professional 4).*

#### Involvement of the family members

It was noted that a relative accompanied almost all the patients. During the informal discussions, it was revealed that the family members play a major role in decision making related to seeking help. The mental health professionals talked about both the positive and negative aspects of the family’s involvement.

*Involvement of the family is good for recovery (mental health professional 14) Family environment and economic position may limit the use of these therapies (mental health professional 15).*

#### Stigma: A significant barrier to help-seeking

The mental health professionals talked about the strong stigma attached to mental illness. Families and relatives of the patients are concerned about people discovering that their relative required treatment from a mental health professional. This hinders the initial help seeking as well as the follow-up visits for therapy.

*The stigma is a big problem; the patients are reluctant to come or even refuse to see psychiatrists (mental health professional 1)*

### Issue related to system and resources

#### Training in CBT for psychosis

None of the mental health professionals had received any formal training in CBT. Although, most of them had some knowledge of CBT and had used the techniques within an eclectic framework. Some of them had some training in rational-emotive behavior therapy (REBT) and psychodynamic therapy.

#### Referral system& pathways to care

Currently, the referral system in China is not highly established. Most patients, their carers, and the mental health professionals talked about self-referral based on a relative or friend’s advice. The participants also described their satisfaction with the online information, registration and appointment system. However, one mental health professional said difficulty in registration is a barrier to receiving help.

*For instance, if a student displays abnormal behaviour, he sent for a psychology teacher. If the psychology teacher realises he has some psychiatric problems the parents are informed, who will bring the student to our hospital (mental health professional 12)? Most of them came to see me without a referral. Some came with the guidance of information online (mental health professional 5). I searched “the top ten hospitals” online. I also called a relative who is an MD student at this hospital (Carer 4)*.

It was evident that the patients follow a complex pathway in their help-seeking journey. It usually starts with seeking help from non-medical healers at the initial stages of the illness. Before coming to the psychiatric health facility almost everyone had seen a traditional/faith healer.

*That guy worked as a magician, he spat fire on me and then said "I am leaving now," but nothing happened (Patient 2). The wizard threw rice grains on the ground, painted mysterious figures on the paper and burned it to ashes; then the ashes were mixed with water and drunk by the patient. It cost us two hours Hundreds of Yuan Renminbi (RMB) (Carer 3)*.

### Understanding of illness, and beliefs about its causes and its management

#### Presenting problems and the effect of schizophrenia

There was a difference in patients’ and carers’ perspective on this. Patients were primarily troubled with the psychotic symptoms while carers sought help because of problematic behaviours. The mental health professionals confirmed this in their interviews. All the patients reported that the illness had affected their lives. The mental health professionals described the following symptoms as presenting complaints by the patients or main concerns by their carers; insomnia, nightmares, retardation of thinking, balderdash, disorganized behaviors, hallucinations, paranoia, excitability, aggression, reduced words and movements, relationship problems, abnormal behaviors, depressed mood, suicide thoughts, and behaviors. These were confirmed by the patients and their carers;

*My classmates persecute me. They dropped my phone. When I came home, I saw one of my classmates was putting feces on my bed. They also spread rumours about me and poisoned my milk. All the patients in the ward would do bad things to me. The boy I like is trying to help me. He controls the minds of other patients to get me out of here. I can hear the thoughts of others. I do not believe other people. My school records are declining (Patient 1). Sometimes when I am alone, I can hear strange voices calling me names and talking bad things about me. I am sure that someone is going to persecute me, but I don’t know where he is. I am afraid to fall asleep at night because that guy could jump out from the dark and kill me quickly. That is why I always feel tired (Patient 2)*.

#### Causes of illness

Most patients did not know the cause of their illness. Some patients and carer related the symptoms to psycho-social problems and stress. Almost all the patients had seen spiritual or alternative healers in the past. However, they did not consider the illness to be due to spiritual causes at the time of the interview. Some of the carers and patients believed in biological causes, such as genetics or disturbances in the brain. In most cases they described multiple causes of illness, as typified by these participants:

*This disease (schizophrenia) is caused by stress, problems in the society and family problems. I was forbidden to meet other people for three days because of their superstitious belief (Patient 1). There are too many stimuli that caused this illness (Patient6). It is caused by too much pressure from studies and outbreak of emotions due to long-term repression (Patient 7). Yes, and psychological stress, trauma, social factors such as being laughed at (Carer 1) Both the genetic factors and the environmental factors. I mean the external environment, like air pollution and the contamination of the ground of the earth (Carer 3)*.

#### Awareness of illness

A significant number of patients and their carers recognized schizophrenia to be a mental illness. Although some of them did not know much about the disease, most had a good awareness of the illness.

*Yes, it is an illness. It is just irritable mood and disordered sleep (Carer 1). Schizophrenia means being unable to deal with stress; accumulation of adverse emotions and genetic factors (Carer 4)*.

#### Knowledge of treatment and expectations

Only a few had heard of psychotherapy. Other did not know what it means. Those who knew of psychotherapy, mainly considered it to be counseling. They expected medical and psychological treatments from the hospital.

*I know a little about it. However, (I think) a comprehensive therapy combining medical therapy, psychotherapy and family therapy is the best option for me (Patient 3). She (the patient) needs a therapy that can cure her disease without the possibility of relapse (Carer3). The psychologist should provide -Psychological guidance, opening the knot of the patient’s mind and help her go out of her small world (Carer 3). It should be efficient and effective. I know the illness cannot be treated completely, only can be controlled by medicine. I wish someday there will be a radical treatment. I would like to add some physical therapy such as ECT. More advanced machines should be used in the treatment (Patient 9).*

### Assessment, engagement & adjustments in therapy

#### Barriers in accessing and delivering therapy

Mental health professionals reported up to 80% drop-out from the follow-up.

They described problems with engagement, high drop outs, lack of awareness of disease and therapy, uncooperative family caregivers or conflicts within the family, dissatisfaction with the therapeutic effect and adverse effect of the drugs.

Traveling distance and traveling expenses were described to be a significant barrier. Mental health professionals said they often follow patients up through phone calls, but they even change their numbers. Nearly all of them said time was a major obstacle in providing and receiving therapy. They often talked about the high volume of patients they have to see in their clinics. They described the lack of time to be the primary constraint in providing therapy to their patients. Surprisingly none of the mental health professionals described homework assignments to be a problem, even on direct questioning. Most of the professionals said that patients expect psychotherapy for non-psychotic disorders. Interviews with both the patients and their carers confirmed this. None of the patients had been offered CBT, although some had received supported counseling. They also talked about the lack of training and availability of therapy. They also talked about patient’s high expectations from treatment. One mental health professional (mental health professional 9) described the difficulty of establishing a collaborative relationship to be a barrier to treatment. They also described other obstacles in providing psycho-social therapies;

*Most of the patients do not have time or finances for long term therapy. Moreover, they usually have a low level of education (mental health professional 13). The standards of this treatment are low; they are likely to turn into health literacy. Moreover, we are short of the systematic psychotherapeutic regime (mental health professional 12). Some think that psychotherapy has an amazing effect, while other believe that it makes no difference (mental health professional 12). At the time of the first admission, the patients and their family members have high expectations in therapeutic response and wish that their diseases can be cured (mental health professional 10). Most people believe that psychotherapy is ineffective and unaffordable (mental health professional 9)*.

#### What works and which techniques help?

During the interviews mental health professionals described the techniques they found helpful in dealing with patients. They reported that socio-economic background plays a significant role in people’s attitude to and therefore their response to psychotherapy. The affluent, educated and urban sections of the population appreciated the value of psychotherapy.

*The low socio-economic level of the patients and their family members is the biggest barrier. Many patients and their family members have difficulty communicating with the doctors, and they do not want to spend time and energy in family therapy or CBT. On the other hand, these methods are not available in most psychiatric hospitals (mental health professional 2). People with high level of education who are dissatisfied with their character (personality issues) but with better compliance usually come for psychotherapy (mental health professional 12)*.

#### The therapeutic relationship

The mental health professionals talked about the importance of the therapeutic relationship and its significance in therapy. For example, these mental health professionals said;

*This (Knowledge and beliefs about health, illness, and health system) may have an effect on the establishment of therapist-patient relationship (mental health professional 1) I found that patience and establishment of the therapeutic relationship are of fundamental importance (mental health professional 8).*

#### Adjustments in therapy

All the health professionals talked about the lack of awareness and education. They suggested that educating patients about illness was the most useful part of treatment. Commonly used techniques that they identified as useful for patients with psychosis included helping with coping, behavioural techniques, social skills, family counseling, dealing with family conflicts. Most of the mental health professionals were not aware of problem solving and Socratic dialogue. One mental health professional who was aware of the Socratic dialogue said it does not work. Most of them said patients expect a directive style rather than collaborative style. They frequently talked about herbal medicine. Only one mentioned including spirituality in treatment.

*As a psychiatrist whose patients mainly have schizophrenia and bipolar affective disorder, the primary purpose of psychotherapy is to improve compliance, to reduce the stigma of disease to increase their social support, and to try to improve the influence on their social function (mental health professional 12). Theoretically speaking, people with religions should be more philosophical, but some so-called religions in China make patients believe in spirits, they may not have a good influence on people. Patients with high level of education have a better acceptation and comprehension of psychotherapy, and therapists with greater knowledge of culture and religions are more efficient (mental health professional 12). The style of therapy here is instructive (mental health professional 11)*.

## Discussion

Patients and their families in China use a bio-psycho-social model of psychosis for conceptualisation and management, with additional emphasis on spiritual and religious causes. This study confirms our previous findings that people from Non-Western cultures use a bio-psycho-social-spiritual model of illness [15]. This is the first study to examine views of the patients, carers and the mental health professionals regarding psychosis from China. While there is one report from China that CBT for psychosis works, the therapy was provided by expert therapists, and additional supervision was given to deal with cultural issues. Findings from this study can help both expert and non-expert therapists in addressing cultural barriers.

The mental health professionals we interviewed in this study emphasized the need for adaptation of treatment and the need to be mindful of cultural, spiritual and other related factors while providing therapy. As identified in previous literature, these findings confirm the need for adapting CBT in non-Western cultures [8, 11, 12, 24]. This study highlights the need for understanding the patient’s cultural and spiritual needs. It also came to fore that the language needs adapting rather than just being translated. It is essential that the therapists in Non-Western countries make adjustments in therapy while keeping the treatment close to its theoretical underpinning. One such example is the involvement of the family. A family member often accompanies patients in China, and mental health professionals insisted on involving the family in decisions regarding the care of patients. Similarly, dealing with stigma in therapy should be an essential part of therapy, as, like the west, it might prevent people from help seeking in LAMIC, but to a greater degree [6, 15, 18].

In the absence of a well-established referral system, clients come from a variety of sources. Self-referral is common. It was evident that the similar to other Asian countries [18] as part of their complex journey patients usually seek help from non-medical healers first. Even though none of the carers or patients considered a spiritual or religious cause, almost all the patients had seen traditional healers or faith/spiritual healers before coming to the psychiatric health facility. The health professionals are seen in high esteem in most Asian countries. It is possible that the patients and their carers were not very comfortable in disclosing their beliefs directly regarding spiritual causes. However, the fact that they had contacted non-medical healers and had spent much money means they believed in these causes at least in the past. It highlights the need to understand the complex pathways used by the patients with psychosis in China. The therapists should adopt an open-minded approach so that patients discuss the involvement of other healers. It might also help understand barriers to receiving help from the modern medical system. Both the carers and the patients knew little about the causes of the illness and in general related the illness to psycho-social problems and stress.

Only a few patients or carers had heard of psychotherapy. It is not surprising, as the mental health professionals were not trained in CBT for psychosis. The mental health professionals were aware of and trained in CBT for non-psychotic disorders, but raised concerns regarding its application in psychosis. They also highlighted difficulties in its application in non-psychotic disorders. Most carers and patients expected medical and psychological treatments from the hospital. It is possible that they see hospitals only responsible for treating them with pills, while their system of mental or spiritual healing exists outside the hospital. It is also possible that only those who are educated, have access to information regarding Western therapies through the internet or are from higher socio-economic background might be familiar with the non-pharmacological interventions. It highlights the need to educate patients.

Similar to our previous work with clients from the non-European background [6] mental health professionals reported a high rate of dropouts from the follow-up. The main problems seem to be with engagement, high drop outs, lack of awareness of disease and therapy, uncooperative family caregivers or conflicts within the family, dissatisfaction with the therapeutic effect and adverse effect of the drugs. Traveling distance and traveling expenses were described to be a major barrier. Surprisingly none of the mental health professionals described homework assignments to be a problem, even on direct questioning. It is possible that clients who see a therapist in a position of authority follow their instructions, as pointed out by Hodges et al. [11].

Interviews with mental health professionals highlighted the techniques that they find helpful in dealing with patients to our attention. Although most of these relate to non-psychotic disorders, with some adjustments, these might be applicable for CBT for psychosis. Our study confirmed the preferable use of a teacher-student relationship instead of collaborative style [8, 11, 14]. Commonly used techniques described to be useful include; helping with coping, behavioural techniques, social skills, family counseling and dealing with family conflicts. The Socratic dialogue was considered a difficult technique to use. It has an intuitive appeal as the model of dialogue in this context in Asian cultures might be sermons delivered by a saint and not a questioning style. It has been suggested that a Socratic questioning style might lead to the patient losing confidence in therapists skills [12, 25].

This study confirms findings from the previous literature, for example, the need for understanding the cultural values, adjustments in techniques to improve assessment and engagement and adjustment in therapy techniques. The lessons learned from this study can be used to deliver culturally sensitive CBT and culturally adapted CBTp.

### Implications for therapy and research

We show through this work that with minor adjustments CBT can be used in China. The information gathered in this study can be easily used to inform “culturally sensitive assessment and formulation to engage clients and adjustments in CBT." An understanding and grasp of the cultural factors will enhance the process of patient engagement with therapy and care.

### Limitations

The mental health professionals interviewed in this project came from a big city and patients and their carers were also mostly city dwellers. This may put some limits on the generalisability of the findings. Including three different groups of participants in the study would have addressed this issue at least partially. Interviews have been criticized over their validity, but in our experience, these are a useful tool in the process of adaptation of CBT as we have shown in our other work cited in this manuscript. An additional point which gives us confidence in the value of interviews is the similarity in the results to our previous work in Pakistan, Morocco and the Middle East and with ethnic minority clients in the United Kingdom.

### Conclusions

This study is part of a bigger body of work focused on adaptation of CBT for non-western countries including China. The information obtained from this study will be used to deliver culturally sensitive CBT for psychosis in a small RCT. We intend to broaden the scope of this work to include patients with other disorders. There is potential to diversify the treatment to wider strata of social and economic sections of population. Finally, there is a need to exploit the full potential of eMedia in this regards.

## Additional file**s**

Additional file 1: CBT with Psychosis Phase I: Patients Interviews. This document describes the questions for the interviews from patients. (DOC 33 kb)

Additional file 2: CBT with Psychosis PHASE II: Relative or care taker’s interview. This document describes questions for interview with the caregivers of the patients. (DOC 32 kb)

Additional file 3: CBT with Psychosis Phase II & III: Psychiatrist & Psychologist’s interviews. This document describes the questions for interviews with professionals (DOC 37 kb)

## Abbreviations

CBT: Cognitive behavior therapy
CBTp: CBT for psychosis
ICD-10, RDC: International classification of diseases, 10th edition, diagnostic criteria for research
LAMIC: Low & middle-income countries
REBT: Rational emotive behavior therapy
RMB: Yuan Renminbi
