# Supplementary material for: A qualitative study to explore views of patients’, carers’ and mental health professionals’ to inform cultural adaptation of CBT for psychosis (CBTp) in China
Source: BMC Psychiatry. 2017 Apr 8;17:131. doi: 10.1186/s12888-017-1290-6 (PMC5385068; doi:10.1186/s12888-017-1290-6)
Supplement: Supplementary file 1 — CBT with Psychosis Phase I: Patients Interviews. This document describes the questions for the interviews from patients. (DOC 33 kb) [file 12888_2017_1290_MOESM1_ESM.doc]

**CBT with Psychosis**

**Phase I: Patients Interviews**

I have to ask you a few questions regarding your illness to know about your ideas and knowledge of your illness. There is no right or wrong answer and your answer will have no effect on your treatment.

Name: _________________________________ Age: _____________ Sex: _________

Education: __________________Marital Status: _________Contact No.:____________

Address: ______________________________________________________________

Diagnosis: ________________________ Duration of Illness: _____________________

Would you please tell me about your problems or symptoms? ____________________

______________________________________________________________________

What do you think of your problem or what it would be? _________________________

______________________________________________________________________

Do you have any illness? _________________________________________________

______________________________________________________________________

Do you know the name of the illness or Is this illness has any name? _______________

______________________________________________________________________

Have you ever heard about schizophrenia (madness, mania)? Is this a physical or mental illness? (Give an example of physical illness if needed as well as a psychological illness that is commonly recognized, for example anxiety, worrying etc.

____________________________________________________________________________________________________________________________________________

How this illness affects your life? Give examples. (Ask for specific areas, work, studies, household chores etc.) ___________________________________________________

______________________________________________________________________

Who referred you or suggested you to come here? _____________________________

______________________________________________________________________

How did they know that your illness can be cured here? _________________________

______________________________________________________________________

What do you think of the reasons of your illness? Additional question, other people have said that it can be because of chemical imbalance, genetics etc. What do u think?

____________________________________________________________________________________________________________________________________________

Have you received treatment from other healers (instead of Doctors)? Like homeopathic doctor, Hakim, Religious Scholar, Magician etc. _______________________________

______________________________________________________________________Do you think, you are in the right place for treatment? And can you be treated well here?_______________________________________________________________________________________________________________________________________

Can doctors cure your illness?_____________________________________________

______________________________________________________________________

How can you get cured of your illness? ______________________________________

______________________________________________________________________What do you know about the treatment of your illness? What are the possible options?

____________________________________________________________________________________________________________________________________________

How should you be treated? With medicines or else? ___________________________

______________________________________________________________________Can you be treated with anything other than medicines? _________________________

______________________________________________________________________

Have you ever heard of psychological treatment/ treatment without medicines/ psychotherapy?_______________________________________________________________________________________________________________________________

Have you ever had the same illness before? __________________________________

______________________________________________________________________

How many previous episodes you have had of this illness?_______________________

______________________________________________________________________

Which treatment you received then? Medical or else? ___________________________

______________________________________________________________________

Who treated you in the past? ______________________________________________

______________________________________________________________________

If you did quit, what was the reason?

____________________________________________________________________________________________________________________________________________

**General questions about mental illness**

Do you know anything about mental illnesses? According to your knowledge, what usually happens in these? What are the symptoms?

____________________________________________________________________________________________________________________________________________

(These are the illnesses which affect the way we think, behave and feel and sometimes about physical symptoms which are not caused by medical illness)

Can you tell us a few names and types of mental illnesses?

____________________________________________________________________________________________________________________________________________

What are the reasons of mental illnesses?

____________________________________________________________________________________________________________________________________________

What do you know about the treatment of mental illnesses?

____________________________________________________________________________________________________________________________________________

Have you ever heard of psychotherapy / talking therapy/ treatment without medicines?

____________________________________________________________________________________________________________________________________________

Who treats mental illnesses?

____________________________________________________________________________________________________________________________________________

Can anyone, other than mental health professional, treat these illnesses?

____________________________________________________________________________________________________________________________________________

What do psychiatrists specialize in?

____________________________________________________________________________________________________________________________________________

Have you ever heard about psychologists?

____________________________________________________________________________________________________________________________________________

Is there anything else you want to discuss or consider important?

____________________________________________________________________________________________________________________________________________
