# Supplementary material for: A qualitative study to explore views of patients’, carers’ and mental health professionals’ to inform cultural adaptation of CBT for psychosis (CBTp) in China
Source: BMC Psychiatry. 2017 Apr 8;17:131. doi: 10.1186/s12888-017-1290-6 (PMC5385068; doi:10.1186/s12888-017-1290-6)
Supplement: Supplementary file 2 — CBT with Psychosis PHASE II: Relative or care taker’s interview. This document describes questions for interview with the caregivers of the patients. (DOC 32 kb) [file 12888_2017_1290_MOESM2_ESM.doc]

**CBT with Psychosis**

**PHASE II: Relative or care taker's interview**

**Demographic Details**

I have to ask you a few questions regarding your patient’s illness to know about your ideas and knowledge of the illness. There is no right or wrong answer and your answer will have no effect on your patient’s treatment.

Name: _________________________________ Age: _____________ Sex: _________

Education: __________________Marital Status: _________Contact No.:____________

Address: ______________________________________________________________

Relation with the patient: _________________________________________________

In your opinion, what has happened to this person? ____________________________

______________________________________________________________________

Do you consider it as an illness? ___________________________________________

______________________________________________________________________

Is he/she needs any treatment for it? ________________________________________

______________________________________________________________________

Is the treatment helpful for the patient? ______________________________________

______________________________________________________________________

Which type of treatment would be better for him/her? ___________________________

______________________________________________________________________How many types of treatment do we have? ___________________________________

______________________________________________________________________

Have you ever heard about psychological treatment/psychotherapy? _______________

______________________________________________________________________Where did you take this patient for the treatment for the very first time? _____________

______________________________________________________________________What was the nature of the illness or treatment? _______________________________

______________________________________________________________________Where did you take this patient for the treatment for the second time? ______________

______________________________________________________________________

What type of treatment did the patient received? _______________________________

______________________________________________________________________

Where did you take this patient for the treatment for the third time? ________________

______________________________________________________________________

What type of treatment did the patient received? _______________________________

______________________________________________________________________Is there anyone else in your family, suffering from this illness?____________________

______________________________________________________________________

**General questions about mental illness**

Do you know anything about mental illnesses? According to your knowledge, what usually happens in these? What are the symptoms?

____________________________________________________________________________________________________________________________________________

(These are the illnesses which affect the way we think, behave and feel and sometimes about physical symptoms which are not caused by medical illness)

What are the names and types of mental illnesses?

____________________________________________________________________________________________________________________________________________

What are the reasons of mental illnesses? ___________________________________ ______________________________________________________________________

What do you know about the treatment of mental illnesses?

____________________________________________________________________________________________________________________________________________

Have you ever heard of psychotherapy / talking therapy/ treatment without medicines?

______________________________________________________________________

______________________________________________________________________

Who treats mental illnesses? ______________________________________________

­­­­­­­­­­­­­­­­­­­­­­______________________________________________________________________

Can anyone, other than mental health professional, treat these illnesses?

____________________________________________________________________________________________________________________________________________

What do psychiatrists specialize in? _________________________________________

______________________________________________________________________

Have you ever heard about psychologists? ___________________________________

______________________________________________________________________

What is the role of a psychologists in the treatment of an illness?__________________

______________________________________________________________________
