# Supplementary material for: A qualitative study to explore views of patients’, carers’ and mental health professionals’ to inform cultural adaptation of CBT for psychosis (CBTp) in China
Source: BMC Psychiatry. 2017 Apr 8;17:131. doi: 10.1186/s12888-017-1290-6 (PMC5385068; doi:10.1186/s12888-017-1290-6)
Supplement: Supplementary file 3 — CBT with Psychosis Phase II & III: Psychiatrist & Psychologist’s interviews. This document describes the questions for interviews with professionals (DOC 37 kb) [file 12888_2017_1290_MOESM3_ESM.doc]

**CBT with Psychosis**

**Phase II & III: Psychiatrist & Psychologist’s interviews**

Name: _________________________________ Age: _____________ Sex: _________

Education: _______________________________ Marital status: _________________

Institute: ______________________________________________________________

Contact no.: ___________________

Which type of patients seen: _______________________________________________

______________________________________________________________________

Which type of therapy provided by you? ______________________________________

______________________________________________________________________

Thank you for taking out sometime and talking to me. Would you like to tell me about your work and work experience? In which specialties have you worked?

____________________________________________________________________________________________________________________________________________

Do you treat patients with psychosis or schizophrenia?

____________________________________________________________________________________________________________________________________________What is the number of patients in your daily practice? What type of problems do they have? ________________________________________________________________

______________________________________________________________________

What is the most common diagnosis? _______________________________________

______________________________________________________________________

Who refers them to you? _________________________________________________

______________________________________________________________________

Can you explain the procedure of their referral in detail? (Either self-referral or referred by others?)_____________________________________________________________

______________________________________________________________________

What are their common symptoms and complaints? ____________________________

______________________________________________________________________

What do they expect of you? ______________________________________________

______________________________________________________________________

How many sessions did you usually take with each patient? ______________________

______________________________________________________________________

What are the common problems people usually have with therapy or other modes of treatment? _____________________________________________________________

______________________________________________________________________
What are the rates of follow-up? (Out of 10) ___________________________________

What are the possible reasons for drop outs? _________________________________

______________________________________________________________________

What are the reasons for poor follow up? _____________________________________

______________________________________________________________________

To what extent treatment related expenses effect the treatment? __________________

______________________________________________________________________

What do people know and think about psychotherapy usually? ____________________

______________________________________________________________________

What do they think about your role in their treatment? ___________________________

______________________________________________________________________

Which other treatment modalities are available? _______________________________

______________________________________________________________________

Do people usually want to take psychotherapy? _______________________________

Which types of people usually come for psychotherapy? _________________________

______________________________________________________________________Can you take me through the steps in therapy, when dealing with these patients? Explain in detail. ________________________________________________________

______________________________________________________________________

Do you use Cognitive Behaviour therapy techniques? ___________________________

______________________________________________________________________

If yes, which one? _______________________________________________________

______________________________________________________________________

Which techniques are effective? ____________________________________________

______________________________________________________________________

Which techniques are not effective? _________________________________________

______________________________________________________________________

Which other techniques do you usually use from other types of psychotherapy?

______________________________________________________________________

Do you want to comment on these areas?

Engagement: __________________________________________________________

Homework: ____________________________________________________________

Therapist-patient relationship: _____________________________________________

______________________________________________________________________

Do you use problem solving? ______________________________________________

For what kind of problems? _______________________________________________

______________________________________________________________________

What is the style of therapy? Instructive style or collaborative: ____________________

______________________________________________________________________Which technique did you usually use to change the thoughts of people? ____________

______________________________________________________________________How do you deal with thoughts? With emotions or with behaviours? ________________

______________________________________________________________________

How therapy in Pakistan is different from the west? _____________________________

______________________________________________________________________Do you think that the therapies, created in west, are a problem to apply in Pakistan?

____________________________________________________________________________________________________________________________________________

What about the use of social skills training? ___________________________________

______________________________________________________________________

Family involvement or family therapy: _______________________________________

______________________________________________________________________

Assertiveness training: ___________________________________________________

______________________________________________________________________

Communication skills: ____________________________________________________

______________________________________________________________________Conflict management: ____________________________________________________

______________________________________________________________________How the following factors effect on therapy?

Religion and Culture: ____________________________________________________

______________________________________________________________________Other factors from the framework: __________________________________________

______________________________________________________________________Organization and structure of the health system: _______________________________

______________________________________________________________________**Patient’s personal factors:**

Knowledge and beliefs about health, illness and health system: ___________________

______________________________________________________________________

Are there any other factors: _______________________________________________

______________________________________________________________________
